# Utility of continuous glucose monitoring for identifying silent hypoglycemia in fructose-1,6-bisphosphatase deficiency: a pilot prospective evaluation

**DOI:** 10.3389/fendo.2025.1664863

**Published:** 2025-10-09

**Authors:** Emine Goksoy, Burcu Kumru Akin

**Affiliations:** ^1^ Division of Pediatric Metabolism, Department of Pediatrics, Gaziantep Cengiz Gökçek Maternity and Children’s Hospital, Gaziantep, Türkiye; ^2^ Division of Nutrition and Dietetics, Gaziantep Cengiz Gökçek Maternity and Children’s Hospital, Gaziantep, Türkiye

**Keywords:** fructose-1,6-bisphosphatase deficiency, continuous glucose monitoring, silent hypoglycemia, uncooked cornstarch, inborn error of metabolism

## Abstract

**Background and objective:**

Fructose-1,6-bisphosphatase (FBPase) deficiency is a rare gluconeogenic disorder characterized by hypoglycemia, lactic acidosis, hyperuricemia, and ketosis, triggered by fasting or infection. Although dietary management aims to prevent hypoglycemia, accurate tools to monitor asymptomatic episodes are lacking. Continuous glucose monitoring (CGM) has not been systematically evaluated in FBPase deficiency. This study aimed to assess the utility of CGM in detecting silent hypoglycemia and its relationship with dietary management.

**Methods:**

Ten genetically confirmed patients underwent blinded CGM using the Medtronic iPro2™ system. CGM metrics included time below range (TBR <70 mg/dL [<3.9 mmol/L]), time in range (TIR 70–150 mg/dL [3.9–8.3 mmol/L]), and time above range (TAR >150 mg/dL [>8.3 mmol/L]). Correlations with biochemical, clinical, and nutritional variables were analyzed using Pearson or Spearman tests, and categorical comparisons were conducted with Fisher’s exact test. Multiple testing was controlled using the Benjamini–Hochberg procedure (significance at FDR-adjusted p<0.05).

**Results:**

Despite using uncooked/modified cornstarch (UCCS/MCS) and frequent feeding (all but one patient), asymptomatic hypoglycemia occurred in some patients. Mean TBR was 11.2 ± 31.2% (Median: 1, Range:0-100). Higher UCCS/MCS dosing correlated with fewer annual metabolic attacks (ρ=−0.854, p-adj=0.002), higher TIR (ρ=0.899, p-adj=0.002), and lower TBR (ρ=−0.917, p-adj=0.003). Patients with TBR≥2% had more annual crises (p=0.003), lower UCCS/MCS dosing frequency (p=0.019), and more hepatic steatosis (p=0.048). Ketonuria correlated with attack frequency (r=0.846, p-adj=0.026). Hepatosteatosis was associated with greater annual attacks (p-adj=0.028).

**Conclusion:**

This, to the best of our knowledge, is the first systematic pilot study of CGM in FBPase deficiency, suggesting a potential role in detecting silent hypoglycemia and informing individualized dietary strategies.

## Introduction

Gluconeogenesis is an important metabolic pathway for glucose homeostasis, especially in the absence of liver glycogen. Fructose-1,6-bisphosphatase (FBPase) deficiency (OMIM: 229700) is a rare autosomal recessive disorder caused by pathogenic variants in the FBP1 gene, leading to impaired gluconeogenesis (1–3).

FBPase deficiency is characterized by recurrent episodes of hypoglycemia, lactic acidosis, hyperuricemia, and ketosis, often associated with prolonged fasting, febrile illness, or excess fructose consumption. Its symptoms in neonates and infants may include irritability, hypotonia, seizures, and may progress to coma. Although most patients are asymptomatic between decompensations and have normal growth and development, some children may develop intellectual disabilities, likely related to recurrent or prolonged hypoglycemia (2–4).

Acute management includes intravenous glucose bolus followed by continuous high-glucose infusion. Correction of acidosis is recommended if metabolic acidosis is present. A universally accepted long-term management consensus has not been established. Frequent feeding schedules and fasting periods shorter than eight hours are commonly accepted. The role of slowly absorbed carbohydrates such as UCCS/MCS during illness or wellness periods remains controversial (3, 4). Diet recommendations vary among centers, but 1-2 g/kg/dose, with a nighttime dose, is more common. Some centers recommend the restriction of fructose, sucrose, and sorbitol, while others consider this necessary only in cases of excessive intake (4). Prevention of fasting-induced hypoglycemia is the primary focus in the management of FBPase deficiency. The main objectives of nutritional management are to avoid hypoglycemia (<70 mg/dL or 4 mmol/L) and complications (3).

Dietary intervention in FBPase deficiency is commonly monitored through capillary glucose measurements and clinical symptoms. However, capillary glucose testing may fail to detect asymptomatic hypoglycemia, thereby delaying appropriate dietary adjustments. Objective tools are needed to manage therapy and investigate new approaches (5–7). In recent years, CGM has been increasingly applied in inherited metabolic diseases (IMDs), including glycogen storage disease and congenital hyperinsulinism with benefit in assessing hypoglycemia and metabolic control. However, its clinical value is still uncertain, and there is debate about its routine use (5–11). In this retrospective system, accuracy was summarized as the mean absolute difference (MAD, %) rather than the mean absolute relative difference (MARD, %). Published evidence indicates reduced accuracy in the hypoglycemic range, and this limitation was considered when interpreting TBR and hypoglycemia burden. Accuracy reporting for retrospective systems such as the Medtronic iPro2/Enlite is variable; in non-diabetic cohorts, studies frequently omit MARD and instead present correlations, mean differences, or MAD% (6). By contrast, Golla et al. quantified MARD for the iPro2/Enlite, reporting an overall value of 8.0% ± 9.2% and demonstrating poorer accuracy in the hypoglycemic range (12).

The purpose of our analysis was to examine the glycemic fluctuations and, in particular, asymptomatic hypoglycemia, using CGM in a cohort of patients with FBPase deficiency. Based on the combination of interstitial glucose data derived from CGM, metabolic control may be optimized and prognosis improved through nutritional and therapeutic recommendations. Monitoring technologies, including CGM, have emerged as important tools in the management of metabolic disorders. Although CGM has been widely used in diabetes and some glycogen storage disorders, there are no prospective data on its use in FBPase deficiency. To the best of our knowledge, this study represents a novel contribution to the literature, as the first systematic assessment of CGM in FBPase deficiency. Although CGM may have implications for improved metabolic assessment and for advanced personalization of care, whether a CGM-guided approach improves longer-term outcomes requires confirmation through prospective, controlled studies.

## Materials and methods

### Study design and participants

This study included 10 patients with genetically confirmed FBPase deficiency who are regularly followed at Gaziantep Cengiz Gokçek Maternity and Children’s Hospital. This study was designed in accordance with the current revision of the Helsinki Declaration and was approved by the local Ethics Committee of Gaziantep University Faculty of Medicine (2020/258) and written informed consent was obtained from each participant and/or guardian. The CGM system was performed by a trained metabolic dietitian, and all participants completed the study with no serious adverse events.

### Continuous glucose monitoring system

CGM systems measure interstitial fluid glucose concentrations every 10 seconds through a subcutaneously inserted sensor coated with glucose oxidase to generate an electrical signal. The system records mean glucose concentrations every 5 minutes, generating 288 daily measurements for continuous follow-up over several consecutive days.

In this study, interstitial glucose concentrations were recorded within a range of 40–400 mg/dL (2.2–22.2 mmol/L) over three consecutive days using CGM in all participants. Detailed dietary logs were maintained concurrently throughout the monitoring period. The CGM system employed was the Medtronic iPro2™ with Enlite™ sensors, which use wireless connectivity and do not require patient calibration. Retrospectively generated glucose control data were interpreted along with biochemical and growth parameters as well as with dietary records to evaluate the success of dietary interventions and to provide required modifications. Unlike the regular 24-hour glucose profile, CGM data capture dynamic glucose trends over time; thus, it provides insight into glucose fluctuations rather than isolated point measurements. Most CGM recordings were blinded using iPro2™ and Enlite™ sensors to detect asymptomatic hypoglycemia independent of symptoms. Both patients and physicians were blinded to glucose measurements during CGM monitoring. A short three day follow-up interval was chosen to balance data collection with feasibility and patient comfort.

During the study period, patients performed capillary blood glucose measurements at least four times daily (before breakfast, dinner, bedtime, and before consuming uncooked cornstarch at night) using glucometers. These values were used for accuracy verification when CGM data were uploaded. For accurate reporting, the Medtronic iPro2 device with an Enlite sensor does not provide MARD. Therefore, we used the device-generated Mean Absolute Difference percentage (MAD%), which is automatically calculated by dividing the difference between the paired sensor glucose and blood glucose (BG) meter readings by the BG meter reading and averaging across all pairs.

Analyzed CGM metrics included time in range (TIR, 70–150 mg/dL [3.9–8.3 mmol/L], %), time below range (TBR, <70 mg/dL [<3.9 mmol/L], %), and time above range (TAR, >150 mg/dL [>8.3 mmol/L], %), with thresholds pre-specified to harmonize with prior non-diabetic metabolic cohorts (7, 9, 10, 13). Parameters investigated in addition to the time metrics included minimum-maximum glucose levels, mean glucose, coefficient of variation (CV%), and Glucose Management Indicator (GMI). All CGM data were analyzed using CareLink iPro Software and reviewed independently by a metabolic dietitian and specialist.

### Dietary management and diet adherence verification

Before the study, dietary regimens were reviewed and adjusted to meet age-appropriate dietary recommendations. UCCS/MCS was prescribed at 1 g/kg/dose with three doses (including a bedtime dose) for at least one month prior to CGM application. Fasting periods during the day were limited to a maximum of 4 hours, and the nighttime UCCS dose was scheduled between 22:00 and 23:00. In our center, dietary plans recommend avoiding excessive fructose intake without restricting sorbitol or fruits. CGM was applied one month after dietary revision and the final check. Before CGM implantation, patients received detailed training on how to record their dietary intake, including the use of standard home measuring devices and/or kitchen scales. Following CGM implantation, patients were asked to keep detailed dietary records for three days. At the end of the three days, these records were reviewed and cross-checked by a dietitian specializing in metabolic diseases, using standard household measures and/or kitchen scales and, when available, photographic documentation provided by the participants to verify portion sizes. All dietary data were analyzed in detail using the Nutrition Information System (BeBIS 8.2) software.

### Patient data collection

Clinical data were abstracted from medical records (demographics, anthropometry, annual metabolic attack frequency) and routine evaluations. Dietary recording and adherence procedures are detailed in the subsection “Dietary Management and Diet Adherence Verification”. Metabolic attack was defined as an acute metabolic decompensation requiring emergency department presentation or hospital admission, accompanied by ≥2 of the following: metabolic acidosis (pH <7.30 or bicarbonate <18 mmol/L), ketosis, elevated lactate (≥3.0 mmol/L), and/or hypoglycemia (plasma glucose <70 mg/dL) (14).

Anthropometric measures were assessed using the WHO Anthro and Anthro Plus software for weight-for-age (WAZ), height-for-age (HAZ), and body mass index (BMI) Z-scores by age and sex. Biochemical parameters measured on the day of CGM placement were obtained in the morning (between 07:00–08:00) following an overnight fast of at least eight hours. These included uric acid (UA), creatinine, lactate, blood gases, triglycerides (TG), alanine aminotransferase (ALT), aspartate aminotransferase (AST), total bilirubin (TBil), direct bilirubin (DBil), gamma-glutamyl transferase (GGT), coagulation parameters (International Normalized Ratio—INR), HbA1c, and urinary ketones. Abdominal ultrasonography (USG) was performed on all patients. The standardized fasting condition was selected to minimize the acute effects of recent food intake on glucose, lactate, ketone bodies, acid–base parameters, and other metabolic variables, and to facilitate comparability between patients. We also standardized the time point (between 07:00–08:00) to reduce the effects of diurnal variation on metabolic parameters.

### Statistical analysis

All statistical analyses were conducted using SPSS software (IBM SPSS Statistics for Windows, Version 18.0. IBM Corp., Armonk, NY). Descriptive statistics were presented as frequencies and percentages for categorical variables and as mean ± standard deviation (SD) for continuous variables. Comparisons between groups were conducted using the Chi-square test or Fisher’s exact test for categorical variables, depending on the expected cell counts. For two-group comparisons of numerical variables, the Student t-test was used when the assumption of normal distribution was satisfied, and the Mann–Whitney U test was applied when this assumption was not met. The degree of relationship between numerical variables was evaluated by Pearson correlation analysis, while monotonic relationships were assessed by Spearman correlation analysis. In the correlation analysis, values between 0.10-0.29 were interpreted as low/weak correlation, 0.30-0.49 as moderate correlation, and 0.50-1.00 as strong correlation. A p-value <0.05 was considered statistically significant. We applied the Benjamini–Hochberg false discovery rate (FDR) procedure at 5% to control multiple testing. Both raw and FDR-adjusted P values (*p-adj*) are reported, with significance defined as *p-adj* < 0.05. Primary analyses used the full cohort (n=10). A pre-specified sensitivity analysis excluding patient P2 was performed for all CGM metrics and correlations; results are provided in **Supplementary Table S2** and did not materially change effect estimates or inference.

## Results

### Study participants

CGM was applied to 10 patients (5 females/5 males) diagnosed with FBPase deficiency from eight unrelated families. The mean age, WAZ, and HAZ of patients were 6.6 ± 3.7 years (min: 2.2; max: 13.2), 0.3 ± 1.4 (min: -1.2; max: 3.1), 0.5 ± 1.6 (min:-2; max: 2.4), respectively. The median BMIZ was 0.45 (0.4-1.2) (min: 0.2; max: 2.6).

In addition to anthropometric measures, biochemical parameters obtained during CGM (AST, ALT, GGT, TG, UA, lactate, TBil, DBil, INR, HbA1c, Ph, bicarbonate-HCO3, urinary ketones), abdominal ultrasonography findings, and annual metabolic decompensation episode frequency were analyzed to comprehensively evaluate the patients’ metabolic status (**Supplementary Material**).

### CGM results

MAD, defined as the average percentage difference between sensor and glucometer readings, was calculated. Differences <28% are considered accurate across all glucose values, while differences <18% are acceptable for glucose levels <100 mg/dL (5.6 mmol/L). In this study, the mean MAD was 10.1 ± 2.7%, indicating that the CGM measurements were within the acceptable accuracy range.

The main CGM results, including mean glucose levels, CV%, GMI%, TIR%, TBR%, and TAR%, are presented in **Table 1**. Compared with healthy pediatric CGM results (15, 16) (**Table 1** footnote), our cohort showed lower TIR (88.1 ± 31.0%) and higher TBR (11.2 ± 31.2%) (Median: 1, Range: 0-100). Different TIR ranges and sensor usage should be considered. In sensitivity analyses excluding patient P2, descriptive metrics were similar overall; notably, TBR decreased substantially, with no change in the direction or significance of inferences (**Supplementary Table S2**). Patients did not report symptoms during hypoglycemia periods, confirming that all were asymptomatic. The mean glucose level was 92.5 ± 16.4 mg/dL [5.14 ± 0.9 mmol/L], with minimum and maximum glucose values of 41 mg/dL and 166 mg/dL (2.3–9.2 mmol/L), respectively.

**Table 1:** CGM-derived glycemic metrics (n=10).

Variables	Mean ± SD
Mean glucose (mg/dL [mmol/L])	92.5 ± 16.4 [5.1 ± 0.9]
TIR (70–150 mg/dL [3.9–8.3 mmol/L]), %	88.1 ± 31.0
TBR (<70 mg/dL [<3.9 mmol/L]), %	11.2 ± 31.2 {Median: 1, Range: 0–100}
TAR (>150 mg/dL [>8.3 mmol/L]), %	0.7 ± 1.2
GMI, glucose management indicator %	4.8 ± 0.6
CV, coefficient of variation %	10.6 ± 3.5
MAD, mean absolute difference %	10.1 ± 2.8
Highest value of glucose (mg/dL [mmol/L])	134.4 ± 30.8 [7.5 ± 1.7]
Lowest value of glucose (mg/dL [mmol/L])]	66.1 ± 14.1 [3.7 ± 0.8]

Conversion: 1 mmol/L = 18 mg/dL (divide mg/dL by 18).

Pediatric CGM benchmarks: In healthy children aged 1–6 y, mean glucose is 103 mg/dL (5.7 mmol/L); median TIR 70–140 mg/dL (3.9-7.8 mmol/L) is 96% (IQR 92–97%); within-individual CV is 17 ± 3%; median TAR >140 mg/dL (7.8mmol/L) is 3.4% (49 min/day); and median TBR <70mg/dL (3.9 mmol/L) is 0.4% (6 min/day) (15). Similar near-normoglycemic profiles are reported in older pediatric cohorts using Dexcom G6 (16). These benchmarks were obtained with Dexcom systems and a 70–140 mg/dL (3.9-7.8mmol/L) range; they are provided to guide interpretation and are not directly comparable to our 70–150 mg/dL (3.9-8.3mmol/L) range and iPro2/Enlite platform.

TIR, time in range; TBR, time below range; TAR, time above range; GMI, glucose management indicator; CV, coefficient of variation; MAD, mean absolute difference. n=10 for all metrics.

Sensitivity analysis excluding patient P2 is provided in **Supplementary Table S2**.

### Relationship between CGM parameters and diet

Patients’ diets were adjusted based on the predefined adherence criteria (see Methods) as specified by the metabolic dietitian. However, it was noted that the patients partially adhered to both dietary recommendations and UCCS/MCS usage protocols. All patients reported that daytime fasting did not exceed 4 hours. Except for patient P2, all received UCCS between 22:00 and 23:00, along with additional doses throughout the day, as detailed in **Table 2**.

**Table 2:** Data on dietary and CGM results of the patients.

	Dietary regimen	UCCS/MCS intake, g/kg/dose	Frequency of daily UCCS/MCS	CHO (%)	Protein (%)	Fat (%)	TIR %	TBR %	TAR %	Mean Glucose*	Min Glucose*	Max Glucose*
P1	UCCS	0.75	2	55	11	34	94	3	3	109 [6.1]	52 [2.9]	161 [8.9]
P2	None	0	0	53	13	36	0	100	0	50 [2.8]	41 [2.3]	60 [3.3]
P3	UCCS	1	3	56.5	11.5	32	100	0	0	104 [5.8]	92 [5.1]	134 [7.4]
P4	UCCS	1	4	58	12	30	100	0	0	93 [5.2]	71 [3.9]	126 [7.0]
P5	UCCS	0.75	3	55	13	32	100	0	0	92 [5.1]	74 [4.1]	119 [6.6]
P6	UCCS	0.9	1	54	12.5	33.5	94	4	2	88 [4.9]	62 [3.4]	163 [9.0]
P7	UCCS	0.9	3	54.5	12.5	33	98	2	0	90 [5]	65 [3.6]	145 [8.0]
P8	MCS	0.8	3	57	13	30	98	0	2	100 [5.6]	72 [4.0]	166 [9.2]
P9	MCS	1	3	57	13.5	29.5	100	0	0	103 [5.7]	75 [4.2]	130 [7.2]
P10	UCCS	0.8	2	54	12	34	97	3	0	96 [5.3]	57 [3.2]	140 [7.8]

UCCS: uncooked cornstarch, MCS: modified cornstarch CHO: carbohydrate, min: minimum; max: maximum.

TIR: Time in Range, TBR: Time Below Range, TAR: Time Above Range.

Thresholds: TIR 70–150 mg/dL [3.9–8.3 mmol/L]; TBR <70 mg/dL [<3.9 mmol/L]; TAR >150 mg/dL [>8.3 mmol/L].

* Values are shown as mg/dL [mmol/L]; conversion uses 1 mmol/L = 18 mg/dL.

UCCS/MCS was prescribed at 1 g/kg/dose with three doses (including a bedtime dose). Dietary regimens were adjusted to meet age-appropriate dietary recommendations. However, none of the patients adhered fully to both dietary recommendations and UCCS/MCS protocols.

Despite receiving bedtime UCCS, patient P1 still exhibited 3% TBR. Patient P2, who declined UCCS despite medical advice and adhered to frequent meals, limiting fasting to under 4 hours, experienced persistent asymptomatic hypoglycemia throughout the day. Although the family attempted to monitor these episodes using capillary blood glucose testing and provided supplemental feeding accordingly, hypoglycemia persisted even postprandially.

**Figure 1:**
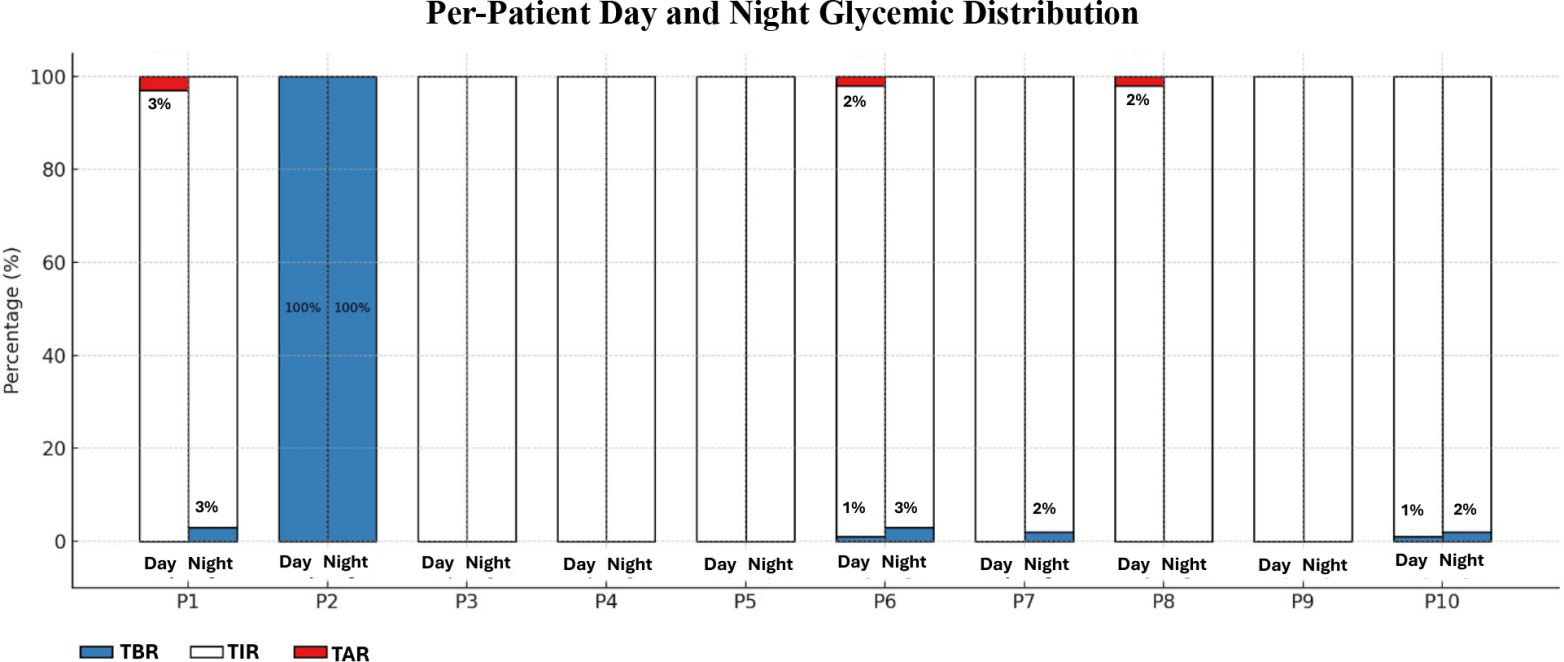
Continuous glucose monitoring-day/night glycemic distribution. Individual distributions of Time Below Range (TBR, <70 mg/dL [<3.9 mmol/L], %), Time In Range (TIR, 70–150 mg/dL [3.9–8.3 mmol/L], %), and Time Above Range (TAR, >150 mg/dL [>8.3 mmol/L], %) during night and day for each patient based on CGM data. Each stacked bar represents the glycemic profile of a patient over a 24-hour period, divided into night (23:00–06:00) and day segments. Blue indicates the percentage of TBR, white indicates TIR, and red indicates TAR. P2 demonstrates persistent hypoglycemia (100% TBR), while P1, P6 and P8 exhibit elevated TAR% values during daytime, despite normal overnight profiles.

Higher frequency of UCCS/MCS correlated with fewer metabolic attacks (ρ=−0.854, p=0.002), higher TIR (ρ=0.899, p=0.001), and lower TBR (ρ=−0.917, p=0.001), but showed no association with TAR (ρ=−0.337, p=0.341) (**Table 3**). Daytime and nighttime TIR, TBR, and TAR values for all patients are presented in **Figure 1**.

**Table 3:** Correlation of UCCS/MCS dose frequency with CGM outcomes and annual frequency of metabolic attacks (n=10).

Variables	Spearman correlation	p-value	p-adj
Annual frequency of metabolic attacks	-0.854	**0.002**	**0,002**
TIR, %	0.899	**0.001**	**0,002**
TBR, %	-0.917	**0.001**	**0,003**
TAR, %	-0.337	0.341	0,341

UCCS, uncooked cornstarch; MCS, modified cornstarch; TIR, time in range; TBR, time below range; TAR, time above range. Thresholds are defined in Methods/**Table 1**. Bold p-values indicate statistical significance (p<0.05). p-adj values were computed using the Benjamini–Hochberg false discovery rate (FDR) procedure across all tests in the table; significance was defined as p-adj < 0.05.

Sensitivity excluding patient P2: **Supplementary Table S2**.

Correlation analysis revealed no significant relationship between glycemic variability and anthropometric indices (HAZ, WAZ, BMIZ). Moreover, there was no significant correlation between glycemic control parameters (CV% and GMI) and the use of UCCS/MCS. A statistically significant, strong negative correlation was observed between the daily UCCS/MCS dose (g/kg) and both the annual frequency of metabolic attacks and TBR. These results suggest that an increase in UCCS/MCS dose is associated with a reduction in annual metabolic attack frequency and time spent below the target glucose range. No statistically significant association was found between the UCCS/MCS dose and either TIR or TAR.

### Group comparison by hypoglycemia burden

**Figure 2:**
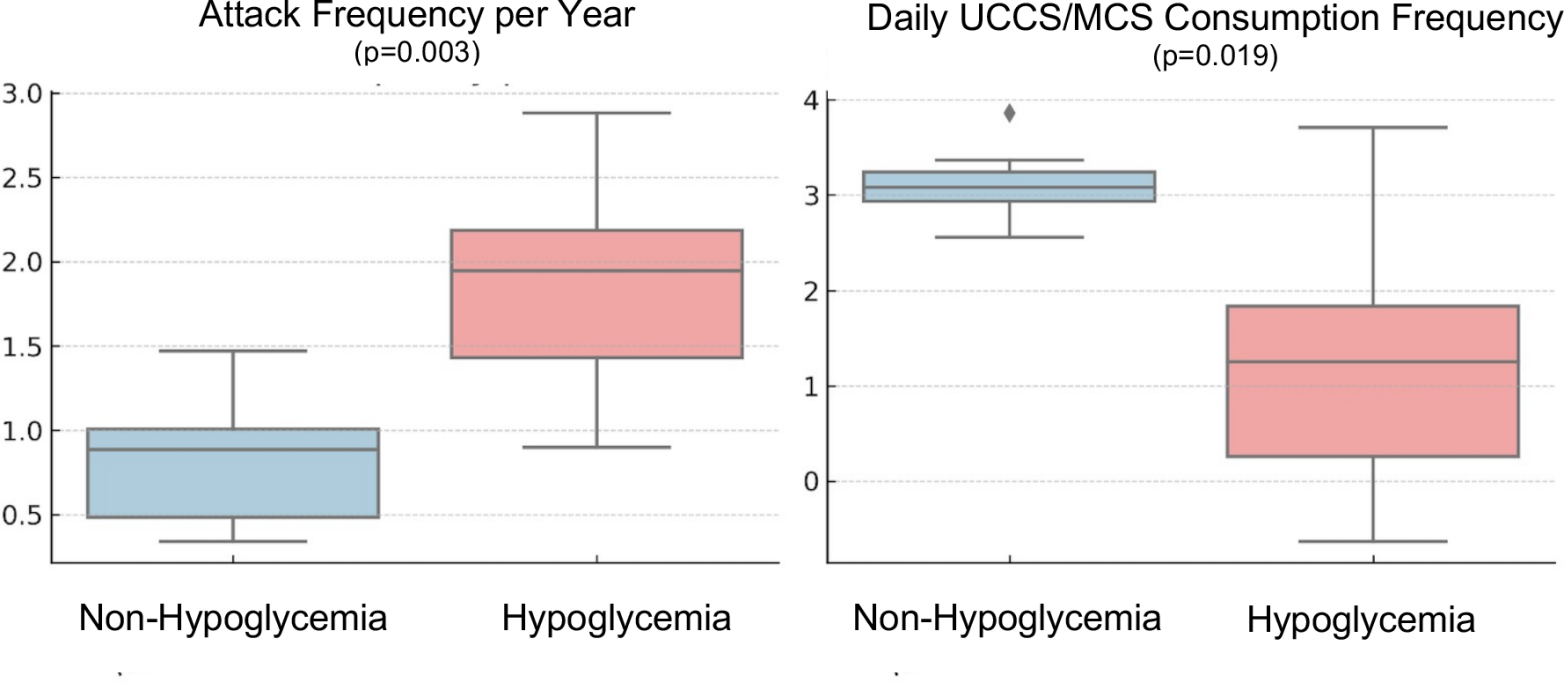
Comparison of annual attack frequency and daily UCCS/MCS dose frequency by hypoglycemia burden (TBR 0% vs ≥2%). TBR, time below range; UCCS, uncooked cornstarch; MCS, modified cornstarch.

Patients were dichotomized by TBR to capture silent hypoglycemia (TBR 0% vs. ≥2%). Compared with the TBR 0% group-non-hypoglycemia group (n=5), the ≥2% group- hypoglycemia group (n=5) had a higher annual attack frequency (2.6 ± 0.9 vs. 0.6 ± 0.6 attacks/year; p=0.003) and a lower daily UCCS/MCS dose frequency (1.6 ± 1.1 vs. 3.2 ± 0.5 doses/day; p=0.019) (**Table 4**) (**Figure 2**). Liver enzymes and other routine biochemistry did not differ significantly between groups (all p>0.05). Hepatic steatosis on abdominal ultrasonography was increased in the hypoglycemia group (80% [4/5] vs. 0% [0/5]; p=0.048). Findings were similar in sensitivity analyses except for patient P2.

**Table 4:** Comparison of clinical, dietary, and laboratory variables by hypoglycemia burden (TBR 0% vs ≥2%).

Variable	TBR 0%	TBR ≥ 2%	p value
Attacks/year	0.6 ± 0.6	2.6 ± 0.9	**0.003**
Daily UCCS/MCS frequency (doses/day)	3.2 ± 0.5	1.6 ± 1.1	**0.019**
AST (IU/L)	31.4 ± 4.3	33.6 ± 10.1	0.667
ALT (IU/L)	23 ± 7.8	33 ± 20.1	0.332
Total bilirubin (mg/dL)	0.43 ± 0.06	0.7 ± 0.37	0.154
Direct bilirubin (mg/dL)	0.15 ± 0.03	0.28 ± 0.12	0.071
GGT (IU/L)	6.8 ± 1.3	12 ± 6.4	0.144
Hepatic steatosis on USG, n/N (%)	0/5 (0%)	4/5 (80%)	**0.048**

TBR %0=non-hypoglycemia group; TBR≥2%=hypoglycemia group.

%TBR, time below range; UCCS, uncooked cornstarch; MCS, modified cornstarch; USG, ultrasonography. Thresholds for CGM metrics are defined in Methods/**Table 1**. Continuous variables are mean ± SD and compared by Student’s t-test; proportions by Fisher’s exact test (two-sided). Bold p-values indicate statistical significance (p<0.05). Sensitivity analysis excluding patient P2 is provided in **Supplementary Table S2**.

### Correlations between laboratory markers, abdominal ultrasonography and glycemic indices

The correlation between attack frequency, glycemic control parameters (GMI, mean glucose, CV), and laboratory markers is summarized in **Table 5**. CV was strongly and positively correlated with ALT, UA, GGT, TBil, and DBil levels. Attack frequency was positively correlated with ketone and bicarbonate (HCO_3_) levels. Because bicarbonate was measured interictally rather than during acute episodes, values were generally normal, accordingly, the small positive correlation with attack frequency is considered exploratory and not clinically meaningful. GMI showed a negative correlation with ketone levels, and mean glucose values were significantly and negatively correlated with ketone concentrations.

**Table 5:** Correlation between annual attack frequency, glycemic indices, and laboratory findings.

Variables	Attack frequency (Attacks/year) r (p) {p-adj}	GMI (%) r (p) {p-adj}	Mean glucose (mg/dl) r (p) {p-adj}	CV (%) r (p) {p-adj}
AST (IU/L)	0.000 (1.000){1.000}	0.307 (0.388){0.769}	0.455 (0.186) {0.546}	0.631 (0.050) {0.108}
ALT (IU/L)	0.200 (0.580){0.641}	0.257 (0.474){0.769}	0.378 (0.281) {0.593}	0,664 (0.036){0.094}
TG (mg/dl)	0.326 (0.358) {0.465}	-0.324 (0.362) {0.769}	-0.435 (0.210) {0.546}	-0.330 (0.351){0.457}
UA (mg/dl)	0.376 (0.285) {0.412}	0.280 (0.434) {0.769}	0.321 (0.365) {0.593}	0.771 (0.009){0.059}
Lactate (mmol/L)	0.438 (0.205) {0.412}	-0.419 (0.228) {0.741}	-0.475 (0.165) {0.546}	0.147 (0.685) {0.685}
Urine Ketone	0.846 **(0.002){0.026}**	-0.668 (0.035){0.455}	-0.650 (0.042){0.546}	0.375 (0.286) {0.457}
TBil (mg/dl)	0.555 (0.096) {0.412}	0.171 (0.636) {0.848}	0.161 (0.656) {0.815}	0.897 **(0.001) {0.013}**
DBil (mg/dl)	0.498 (0.143) {0.412}	-0.097 (0.790) {0.891}	0.022 (0.952) {0.954}	0.714 (0.020){0.065}
INR	0.418 (0.230) {0.412}	-0.163 (0.652) {0.848}	-0.145 (0.690) {0.815}	0.300 (0.400) {0.473}
GGT (IU/L)	0.403 (0.248) {0.412}	0.020 (0.955) {0.955}	0.161 (0.657) {0.815}	0.735 (0.015){0.065}
pH	0.194 (0.591) {0.641}	-0.421 (0.226) {0.741}	-0.340 (0.337) {0.593}	0.187 (0.605) {0.656}
HCO3	0.753 (0.012){0.078}	-0.523 (0.121) {0.741}	-0.480 (0.160){0.546}	0.330 (0.351) {0.457}
HbA1c (%)	0.380 (0.278) {0.412}	-0.082 (0.822) {0.891}	0.021 (0.954) {0.954}	0.429 (0.216) {0.401}

AST, aspartate aminotransferase; ALT, alanine aminotransferase; TG, triglycerides; UA, uric acid; TBil, total bilirubin; DBil, direct bilirubin; GGT, gamma-glutamyl transferase; INR, international normalized ratio; HCO_3_, bicarbonate.

All correlation coefficients (r) were calculated using the Pearson correlation test. Adjusted p-values (p-adj) were calculated using the Benjamini–Hochberg false discovery rate (FDR) procedure across all tests in the table; statistical significance was set at p-adj < 0.05. Attack frequency showed a strong positive correlation with urine ketone (r = 0.846, p-adj = 0.026). CV% demonstrated a positive correlation with total bilirubin (TBil) (r = 0.897, p-adj = 0.013). However, the clinical relevance is uncertain. This exploratory finding should be interpreted with caution and has no immediate clinical implications. No correlations involving mean glucose reached significance, and the remaining correlations were not significant by the FDR criterion (p-adj ≥ 0.05). Bold values indicate statistical significance at p < 0.05.

When comparing annual attack frequency with abdominal ultrasonography (USG) findings, patients with normal USG findings had significantly fewer metabolic decompensation episodes compared to those with hepatic steatosis (p=0.007). However, there was no significant difference between these groups in terms of glycemic control parameters (GMI, mean glucose, glycemic variability), as shown in **Table 6**.

**Table 6:** Comparison of Abdominal USG Findings and Glycemic Control Parameters.

	Abdominal USG		p-value	p-adj
	Normal	Hepatosteatosis		
Attacks/Yr:	0.8 ± 0.8	2.8 ± 1	0.007*	0.028*
GMI (%)	5.0 ± 0.3	4.6 ± 0.9	0.385	0.385
Mean Glucose (mg/dL [mmol/L])	97 ± 6.1 [5.4 ± 0.3]	85.8 ± 25.4 [4.8 ± 1.4]	0.315	0.385
CV%	9.6 ± 1.9	12.2 ± 5.1	0.290	0.385

USG, Ultrasonography; Attacks/Yr, The number of metabolic decompensation episodes per year.

GMI, Management Indicator; CV%, coefficient of variation.

p-adj values were computed using the Benjamini–Hochberg false discovery rate (FDR) procedure across all tests in the table; significance was defined as p-adj < 0.05. Bold values indicate statistical significance at p < 0.05.

## Discussion

This study provides the first assessment of CGM in patients with FBPase deficiency and contributes to the growing use of CGM in non-diabetic IMDs (6, 7, 13). CGM provides valuable insight into asymptomatic glycemic fluctuations, playing a critical role in the management of dietary and medical interventions for IMDs with hypoglycemia. The device-reported accuracy metric was MAD%, which in our study was 10.1 ± 2.7%. MAD indicates the high accuracy of CGM by the values less than 28% (17).

Prior GSD studies likewise documented prolonged asymptomatic hypoglycemia using CGM (5, 7–10, 18). In some of these studies, alarm-enabled systems were used, whereas in others alarms were disabled to preserve observational validity. While our retrospective, blinded Medtronic iPro2 system without real-time monitoring may be regarded as a limitation; however, it enabled observation of the unaltered natural course of glucose fluctuations in FBPase deficiency by avoiding behavioral changes triggered by alarms. This approach is widely used in observational CGM studies designed to detect silent hypoglycemia rather than intervene, thus allowed assessment of true TBR performance (9, 18). In addition to device selection, the applied TBR thresholds also vary, with some studies adopting values <54 mg/dL or <50 mg/dL (13). In our study, consistent with prior GSD reports (10, 16), the TBR threshold was set at <70 mg/dL. However, applying multiple thresholds (e.g., <70, <54, <50 mg/dL) may allow for more precise grading of silent hypoglycemia and provide greater clinical benefit in guiding treatment decisions.

Future studies should aim to utilize CGM devices with improved accuracy for hypoglycemia (i.e. low MARD) and platforms that would allow multiple threshold analysis. This will enhance accuracy for detecting silent hypoglycemia and will assist in customizing individual dietary and therapy regimens. It will also expand the application of CGM monitoring in FBPase deficiency as well as related metabolic disorders.

Although nutritional management in FBPase deficiency is not well defined, preventing hypoglycemia by frequent carbohydrate intake and avoiding prolonged fasting remains the primary goal. Although fructose is a gluconeogenic precursor, the necessity and degree of sucrose and fructose restriction remain controversial (4). Modified cornstarch (MCS) has been proposed as a superior alternative due to its slower release profile, but in our study, neither UCCS nor MCS correlated with CV% or GMI, consistent with findings in hepatic GSDs that metabolic benefits arise from CGM-guided adjustments regardless of carbohydrate source (9).

In a multi-center study of 126 patients, dietary practice was highly variable, and most centers recommended some restriction of sucrose/fructose, but only 37% of patients received UCCS (4). Despite limited adherence observed in this cohort, a significant negative correlation was observed between UCCS/MCS doses and both TBR and annual attack frequency, highlighting the value of bedtime supplementation ≥1 g/kg/dose (**Table 3**). Notably, when stratified by TBR, patients with silent hypoglycemia (TBR ≥2%) received lower effective doses and had higher attack rates, underscoring the therapeutic relevance of adequate starch therapy (**Table 4**). Conversely, non-hypoglycemic patients achieved stable control, in some cases with 100% TIR, despite variable adherence. Supporting the need for vigilant monitoring, Åsberg et al. reported recurrent hypoglycemia up to age 10 (19). Similarly, One untreated 11-year-old patient (P2) exhibited persistent hypoglycemia during CGM (41–60 mg/dL), challenging earlier reports of improved fasting tolerance with age (2, 20). By contrast, some younger patients achieved TIR of 100%, while others showed nocturnal asymptomatic TBR values of 2–4%. These findings highlight the highly individualized nature of therapy in FBPase deficiency and the key role of CGM in tailoring dietary strategies.

There is no published guidance on glucose monitoring in FBPase deficiency. Patients in this cohort did not routinely perform capillary blood glucose monitoring and only check glucose levels when symptoms arise. Given that our study identified asymptomatic hypoglycemia despite treatment, we indicate that intermittent CGM may be warranted to assess fasting tolerance in these patients. In settings where CGM is unavailable, periodic capillary blood glucose monitoring, including nocturnal measurements, may be warranted, albeit less intensively than in diabetic patients.

In our study, no significant associations were observed between CGM metrics and routine biochemical parameters, except for urine ketone positivity and total bilirubin. Most prior work (5, 8, 9, 21, 22) has focused on the ability of CGM to detect hypoglycemia and enable timely intervention, and there are few studies that compare these parameters cross-sectionally. A study in patients with GSD Ia reported that biochemical markers did not represent dynamic glycemic changes (18). Worth et al. noted that sensitivity for detecting hypoglycemia may be lower in non-diabetic cohorts, which may undermine the relationship between simultaneous capillary/venous glucose and CGM measurements (6). Although we were unable to measure MARD (mean absolute relative difference) under hypoglycemia in our dataset, Golla et al. found a high MARD, which is associated with lower sensitivity for hypoglycemia detection with the same device (12). This could explain the weak relationships between TBR-derived CGM metrics and biochemical parameters in our cohort. In addition, CV% showed a positive correlation with total bilirubin; however, this exploratory finding should be interpreted cautiously, as its clinical relevance remains uncertain.

Although ketotic hypoglycemia is frequently mentioned in FBPase deficiency, detailed ketone data are scarce. Dalili et al. reviewed 104 reported cases and noted only two with documented ketone positivity during the acute attack without follow-up data (23). In our study, we found a strong correlation between interictal urine ketone positivity and attack frequency. Given that urinary ketone levels are obtained after approximately 8 hours of fasting, it is possible that interictal ketonuria reflects reduced fasting tolerance and an underlying tendency toward ketogenesis. CGM studies in GSDs have also shown that the combination of CGM and home ketone monitoring may improve metabolic control and support ketone monitoring as a useful secondary marker in clinical follow-up (6, 21). These results should be confirmed in larger patient studies.

Our study found an association between abdominal ultrasound findings and the frequency of metabolic attacks. In patients who had hepatic steatosis, the rate of metabolic attacks was higher than those with normal USG findings, demonstrating a potential link with liver involvement and disease progression.

Although glycemic metrics derived from CGM were similar in those without and with hepatic steatosis, the higher rate of metabolic attack in patients with steatosis warrants further exploration of the effect of liver pathology on glucose homeostasis. Future studies need to characterize the nature of interactions involving hepatic dysfunction and glucose dysregulation in patients with FBPase deficiency, particularly with long-term outcomes and treatment responses.

## Conclusion

This, to the best of our knowledge, is the first systematic evaluation of CGM in patients with FBPase deficiency, focusing on its use for the detection of silent hypoglycemia and on dietary and therapeutic strategies. CGM may be considered more integrative and comprehensive than standard intermittent glucose monitoring in detecting nighttime and asymptomatic hypoglycemia. Furthermore, CGM may represent a helpful way to evaluate an individual’s fasting tolerance to support personalized management.

## Limitations and future directions

Despite the strengths of our study, there are also limitations. This single-center study had a small sample and a brief 3-day CGM use. This period was kept short to maximize feasibility and patient comfort in a resource-limited pediatric setting, where repeated visits for sensor insertion/removal would have limited participation. We used the retrospective, blinded iPro2/Enlite platform; although the absence of real-time alarms can be viewed as a limitation, in this context, it minimized behavioral reactivity and enabled characterization of silent hypoglycemia under usual dietary practice. Concurrently, iPro2/Enlite has reduced low-glucose accuracy; therefore, accuracy was summarized with device-generated MAD% (not MARD) and TBR estimates were interpreted cautiously. The hypoglycemia threshold was pre-specified at <70 mg/dL to harmonize with prior non-diabetic cohorts and the platform’s default outputs, but future studies should report multi-threshold metrics (e.g., <63 and <54 mg/dL) and consider real-time CGM systems with validated performance in the low range. Despite structured training and verification, adherence to UCCS/MCS varied, introducing heterogeneity. Finally, comparisons with healthy pediatric CGM benchmarks rely on different sensors and ranges (Dexcom, 70–140 mg/dL), which limits cross-platform generalizability. To verify our findings and develop standardized CGM protocols for FBPase deficiency, larger prospective studies are needed with longer follow-up duration.

## Data Availability

The original contributions presented in the study are included in the article/**Supplementary Material**. Further inquiries can be directed to the corresponding author.

## Supplementary material

Supplementary Table 1 Clinical, Biochemical, and Abdominal Ultrasound Findings. Normal reference ranges: AST: (13-40 IU/L for 2-60 years), ALT: (7-40 IU/L for 2-60 years), TG: (normal: <150 mg/dL, Borderline high: 150-199 mg/dL, High: 200-499 mg/dL, Very high: >500 mg/dL), UA: (2.0-5.5 mg/dL for <12 years, 2.3-7.6 mg/dL for adults), Lactate: (<2 mmol/L for children), HbA1c: (4.5-6.5% for children), HCO_3:_ (22-26 mEq/L for children), Urine Ketone: (negative/trace/+1 to +3; positivity ≥+1), TBil: (0.3-1.2 mg/dL for children), DBil: (0.01-0.3 mg/dL for children), INR: (0.8-1.2 for children), GGT: (1-29 IU/L for children). (Wu, A. H. B. (Ed.). (2006). Tietz Clinical Guide to Laboratory Tests (4th ed.). Saunders/Elsevier). Urine ketone: semiquantitative dipstick (negative/trace/+1 to +3). P: Patient, AST:aspartate aminotransferase, ALT: alanine aminotransferase, TG: triglycerides, UA:uric acid, TBil:total bilirubin, DBil:direct bilirubin, GGT:gamma-glutamyl transferase, INR: International Normalized Ratio. *Attacks/Yr: The number of metabolic decompensation episodes per year. Abd USG, Abdominal ultrasonography; HS, hepatosteatosis.

Supplementary Table 2 CGM metrics and Glycemic outcomes with daily UCCS/MCS frequency, with and without patient P2. **(A)** Descriptive CGM metrics (mean ± SD). **(B)** Correlation analysis between daily UCCS/MCS frequency and annual attack frequency, TBR, TIR, and TAR. **(C)** Sensitivity analysis with patient P2 and excluding patient P2: comparison by hypoglycemia burden (TBR 0% vs ≥2%). TBR %0=non-hypoglycemia group; TBR≥2%=hypoglycemia group. %TBR, time below range; UCCS, uncooked cornstarch; MCS, modified cornstarch; USG, ultrasonography. Thresholds for CGM metrics are defined in Methods/**Table 1**. Continuous variables are mean ± SD and compared by Student’s t-test; proportions by Fisher’s exact test (two-sided). Bold p values p<0.05. **(D)** Abdominal ultrasonography subgroups and outcomes (mean ± SD). *With Patient 2, **Without Patient 2. Values under A are reported as mean ± SD (from cohort-level tables). Spearman’s ρ in B were computed from patient-level data extracted from the manuscript’s per-patient table. Outcomes: TBR (%), <70mg/dL [<3.9mmol/L], TIR (%), 70–150mg/dL [3.9–8.3mmol/L], TAR (%), >150mg/dL [>8.3mmol/L]. UCCS/MCS, daily frequency of uncooked/modified cornstarch administration (episodes/day). ‘With P2’ includes all patients (n=10); ‘Without P2’ excludes the outlier case (n=9). USG: Ultrasonography, Attacks/Yr: The number of metabolic attack per year. GMI: Management Indicator, CV%: coefficient of variation.

## References

[1] Hanson RW Owen OE. Gluconeogenesis. In: Lennarz WJ Lane MD, editors. Encyclopedia of Biological Chemistry, 2nd ed. Elsevier, Oxford (2013). p. 381–6. doi: 10.1016/B978-0-12-378630-2.00040-2

[2] Mayatepek E Hoffmann B Meissner T. Inborn errors of carbohydrate metabolism. Best Pract Res Clin Gastroenterol. (2010) 24:607–18. doi: 10.1016/j.bpg.2010.07.012, PMID: 20955963

[3] Bijarnia-Mahay S Bhatia S Arora V Adam MP Feldman J Mirzaa GM. Fructose-1,6-bisphosphatase deficiency. In: Adam MP Mirzaa GM Pagon RA Wallace SE Bean LJH Gripp KW, editors. GeneReviews^®^ . University of Washington, Seattle, Seattle (WA (1993–2019). Available online at: https://www.ncbi.nlm.nih.gov/books/NBK550349/. 31804789

[4] Pinto A Alfadhel M Akroyd R Atik Altınok Y Bernabei SM Bernstein L. International practices in the dietary management of fructose 1-6 biphosphatase deficiency. Orphanet J Rare Dis. (2018) 13:158. doi: 10.1186/s13023-018-0760-3, PMID: 29370874 PMC5785792

[5] Maran A Crepaldi C Avogaro A Catuogno S Burlina A Poscia A. Continuous glucose monitoring in conditions other than diabetes. Diabetes Metab Res Rev. (2004) 20:S50–5. doi: 10.1002/dmrr.518, PMID: 15551341

[6] Worth C Hoskyns L Salomon-Estebanez M Nutter PW Harper S Derks TGJ. Continuous glucose monitoring for children with hypoglycemia: Evidence in 2023. Front Endocrinol (Lausanne). (2023) 14:1116864. doi: 10.3389/fendo.2023.1116864, PMID: 36755920 PMC9900115

[7] Gugelmo G Maines E Boscari F Lenzini L Fadini GP Burlina A. Continuous glucose monitoring in patients with inherited metabolic disorders at risk for Hypoglycemia and Nutritional implications. Rev Endocr Metab Disord. (2024) 25(5):897–910. doi: 10.1007/s11154-024-09903-y, PMID: 39352577 PMC11470883

[8] Hershkovitz E Rachmel A Ben-Zaken H Phillip M. Continuous glucose monitoring in children with glycogen storage disease type I. J Inherit Metab Dis. (2001) 24:863–9. doi: 10.1023/A:1013996325720, PMID: 11916320

[9] Peeks F Hoogeveen IJ Feldbrugge RL Burghard R de Boer F Fokkert-Wilts MJ. A retrospective in-depth analysis of continuous glucose monitoring datasets for patients with hepatic glycogen storage disease: Recommended outcome parameters for glucose management. J Inherit Metab Dis. (2021) 44:1136–50. doi: 10.1002/jimd.12383, PMID: 33834518 PMC8519135

[10] Kasapkara CS Cinasal Demir G Hasanoğlu A Tümer L. Continuous glucose monitoring in children with glycogen storage disease type i. Eur J Clin Nutr. (2014) 68:101–5. doi: 10.1038/ejcn.2013.186, PMID: 24149443

[11] Anık A Türkmen MK Akcan AB Ünüvar T Öztürk S Anık A. Experience with real-time continuous glucose monitoring in newborns with congenital hyperinsulinemic hypoglycemia. Z Geburtshilfe Neonatol. (2021) 225:155–60. doi: 10.1055/a-1209-3861, PMID: 32746476

[12] Golla KK Gupta Y Goyal A Kalaivani M Kachhawa G Kulshrestha V. Comparison of accuracy of freestyle libre pro and medtronic iPro2 continuous glucose monitoring systems in pregnancy. Diabetes Technol Ther. (2023) 25:538–42. doi: 10.1089/dia.2023.0070, PMID: 37129276

[13] Klonoff DC Nguyen KT Xu NY Gutierrez A Espinoza JC Vidmar AP. Use of continuous glucose monitors by people without diabetes: an idea whose time has come? J Diabetes Sci Technol. (2023) 17:1686–97. doi: 10.1177/19322968221110830, PMID: 35856435 PMC10658694

[14] Rossi A Hoogeveen IJ Lubout CMA de Boer F Fokkert-Wilts MJ Rodenburg IL. A generic emergency protocol for patients with inborn errors of metabolism causing fasting intolerance: A retrospective, single-center study and the generation of www.emergencyprotocol.net. J Inherit Metab Dis. (2021) 44:1124–35. doi: 10.1002/jimd.12386, PMID: 33844307 PMC8518720

[15] Dubose SN Kanapka LG Bradfield B Sooy M Beck RW Steck AK. Continuous glucose monitoring profiles in healthy, nondiabetic young children. J Endocr Soc. (2022) 6:1–7.. doi: 10.1210/jendso/bvac060, PMID: 35506147 PMC9049110

[16] Shah VN Dubose SN Li Z Beck RW Peters AL Weinstock RS. Continuous glucose monitoring profiles in healthy nondiabetic participants: A multicenter prospective study. J Clin Endocrinol Metab. (2019) 104:4356–64. doi: 10.1210/jc.2018-02763, PMID: 31127824 PMC7296129

[17] Blevins TC. Professional continuous glucose monitoring in clinical practice. J Diabetes Sci Technol. (2010) 4:440–56. doi: 10.1177/193229681000400226, PMID: 20307406 PMC2864181

[18] Rossi A Venema A Haarsma P Feldbrugge L Burghard R Rodriguez-Buritica D. A prospective study on continuous glucose monitoring in glycogen storage disease type Ia: toward glycemic targets. J Clin Endocrinol Metab. (2022) 107:E3612–23. doi: 10.1210/clinem/dgac411, PMID: 35786777 PMC9387687

[19] Åsberg C Hjalmarson O Alm J Martinsson T Waldenström J Hellerud C. Fructose 1,6-bisphosphatase deficiency: Enzyme and mutation analysis performed on calcitriol-stimulated monocytes with a note on long-term prognosis. J Inherit Metab Dis. (2010) 33:Suppl 3:S113-21. doi: 10.1007/s10545-009-9034-5, PMID: 20151204

[20] Moses SW Bashan N Flasterstein BF Rachmel A Gutman A. Fructose-1,6-diphosphatase deficiency in Israel. Isr J Med Sci. (1991) 27:1–4., PMID: 1995492

[21] White FJ Jones SA. The use of continuous glucose monitoring in the practical management of glycogen storage disorders. J Inherit Metab Dis. (2011) 34(3):631–42. doi: 10.1007/s10545-011-9335-3, PMID: 21556835

[22] Overduin RJ Venema A Lubout CMA Fokkert-Wilts MJ De Boer F Schreuder AB. Continuous glucose monitoring metrics in people with liver glycogen storage disease and idiopathic ketotic hypoglycemia: A single-center, retrospective, observational study. Mol Genet Metab. (2024) 143(1-2):108573. doi: 10.1016/j.ymgme.2024.108573, PMID: 39243574

[23] Dalili S Sedighi Pirsaraei N Sharifi A Pouryousef A Aghaee F Bayat R. Intrafamilial phenotypic variability due to a missense pathogenic variant in FBP1 gene. Mol Genet Metab Rep. (2024) 41:101136. doi: 10.1016/j.ymgmr.2024.101136, PMID: 39282051 PMC11402249

