# Supplementary material for: Utility of continuous glucose monitoring for identifying silent hypoglycemia in fructose-1,6-bisphosphatase deficiency: a pilot prospective evaluation
Source: Front Endocrinol (Lausanne). 2025 Oct 9;16:1664863. doi: 10.3389/fendo.2025.1664863 (PMC12545126; doi:10.3389/fendo.2025.1664863)
Supplement: Supplementary Table 1 — Clinical, Biochemical, and Abdominal Ultrasound Findings. Normal reference ranges: AST: (13-40 IU/L for 2-60 years), ALT: (7-40 IU/L for 2-60 years), TG: (normal: <150 mg/dL, Borderline high: 150-199 mg/dL, High: 200-499 mg/dL, Very high: >500 mg/dL), UA: (2.0-5.5 mg/dL for <12 years, 2.3-7.6 mg/dL for adults), Lactate: (<2 mmol/L for children), HbA1c: (4.5-6.5% for children), HCO3: (22-26 mEq/L for children), Urine Ketone: (negative/trace/+1 to +3; positivity ≥+1), TBil: (0.3-1.2 mg/dL for children), DBil: (0.01-0.3 mg/dL for children), INR: (0.8-1.2 for children), GGT: (1-29 IU/L for children). (Wu, A. H. B. (Ed.). (2006). Tietz Clinical Guide to Laboratory Tests (4th ed.). Saunders/Elsevier). Urine ketone: semiquantitative dipstick (negative/trace/+1 to +3). P: Patient, AST:aspartate aminotransferase, ALT: alanine aminotransferase, TG: triglycerides, UA:uric acid, TBil:total bilirubin, DBil:direct bilirubin, GGT:gamma-glutamyl transferase, INR: International Normalized Ratio. *Attacks/Yr: The number of metabolic decompensation episodes per year. Abd USG, Abdominal ultrasonography; HS, hepatosteatosis. [file DataSheet1.pdf]

Supplementary Table S1: Clinical, Biochemical, and Abdominal Ultrasound Findings

	Age (years)	*Attacks /Yr	AST IU/L	ALT IU/L	TG mg/dL	UA mg/dL	Lactate mmol/L	HbA1c %	Ph	HCO_3 mEq/L	Urine Ketone	TBil mg/dL	DBil mg/dL	INR	GGT IU/L	Abd USG
P1	13.2	3	45	55	82	7.8	3	5.27	7.37	23.9	+2	1.36	0.4	1.8	19	HS
P2	11	4	24	11	180	2.3	4	5	7.39	24.3	+3	0.6	0.2	1.7	7	HS
P3	2.6	0	30	18	149	2.3	2	4.9	7.34	21.3	0	0.37	0.11	1.65	5	normal
P4	2.2	0	36	21	45	2.3	3	4.9	7.36	21.2	+1	0.47	0.18	1.3	8	normal
P5	7.4	1	27	37	150	4.8	1.1	5.3	7.4	24	+1	0.48	0.12	1.14	7	normal
P6	5.6	2	44	50	82	4.6	2.4	5.27	7.34	23.9	+2	0.55	0.4	1.8	19	HS
P7	3.4	2	26	14	175	2.2	2.6	4.9	7.39	22	+2	0.5	0.3	1.6	8	normal
P8	4.4	1	28	18	149	2.4	2.5	4.8	7.3	21.3	0	0.37	0.16	1.65	6	normal
P9	9.6	1	36	21	50	2.3	2.8	4.9	7.36	21.2	0	0.47	0.18	1.3	8	normal
P10	6.5	2	29	35	150	4.8	1.1	5.3	7.25	23	+2	0.48	0.12	1.14	7	HS

Normal reference ranges: AST: (13-40 IU/L for 2-60 years), ALT: (7-40 IU/L for 2-60 years), TG: (normal: <150 mg/dL, Borderline high: 150-199 mg/dL, High: 200-499 mg/dL, Very high: ≥500 mg/dL), UA: (2.0-5.5 mg/dL for <12 years, 2.3-7.6 mg/dL for adults), Lactate : (<2 mmol/L for children), HbA1c: (4.5-6.5% for children), HCO_3: (22-26 mEq/L for children), Urine Ketone: (negative/trace/+1 to +3; positivity ≥+1), TBil : (0.3-1.2 mg/dL for children), DBil: (0.01-0.3 mg/dL for children), INR: (0.8-1.2 for children), GGT: (1-29 IU/L for children). (Wu, A. H. B. (Ed.). (2006). Tietz Clinical Guide to Laboratory Tests (4th ed.). Saunders/Elsevier)

Urine ketone: semiquantitative dipstick (negative/trace/+1 to +3)

P: Patient, AST:aspartate aminotransferase, ALT: alanine aminotransferase, TG: triglycerides, UA:uric acid, TBil:total bilirubin, DBil:direct bilirubin, GGT:gamma-glutamyl transferase, INR: International Normalized Ratio

*Attacks/Yr: The number of metabolic decompensation episodes per year.

Abd USG: Abdominal ultrasonography HS: hepatosteatosis
