# Supplementary material for: Utility of continuous glucose monitoring for identifying silent hypoglycemia in fructose-1,6-bisphosphatase deficiency: a pilot prospective evaluation
Source: Front Endocrinol (Lausanne). 2025 Oct 9;16:1664863. doi: 10.3389/fendo.2025.1664863 (PMC12545126; doi:10.3389/fendo.2025.1664863)
Supplement: Supplementary Table 2 — CGM metrics and Glycemic outcomes with daily UCCS/MCS frequency, with and without patient P2. (A) Descriptive CGM metrics (mean ± SD). (B) Correlation analysis between daily UCCS/MCS frequency and annual attack frequency, TBR, TIR, and TAR. (C) Sensitivity analysis with patient P2 and excluding patient P2: comparison by hypoglycemia burden (TBR 0% vs ≥2%). TBR %0=non-hypoglycemia group; TBR≥2%=hypoglycemia group. %TBR, time below range; UCCS, uncooked cornstarch; MCS, modified cornstarch; USG, ultrasonography. Thresholds for CGM metrics are defined in Methods/ Table 1 . Continuous variables are mean ± SD and compared by Student’s t-test; proportions by Fisher’s exact test (two-sided). Bold p values p<0.05. (D) Abdominal ultrasonography subgroups and outcomes (mean ± SD). *With Patient 2, **Without Patient 2. Values under A are reported as mean ± SD (from cohort-level tables). Spearman’s ρ in B were computed from patient-level data extracted from the manuscript’s per-patient table. Outcomes: TBR (%), <70mg/dL [<3.9mmol/L], TIR (%), 70–150mg/dL [3.9–8.3mmol/L], TAR (%), >150mg/dL [>8.3mmol/L]. UCCS/MCS, daily frequency of uncooked/modified cornstarch administration (episodes/day). ‘With P2’ includes all patients (n=10); ‘Without P2’ excludes the outlier case (n=9). USG: Ultrasonography, Attacks/Yr: The number of metabolic attack per year. GMI: Management Indicator, CV%: coefficient of variation. [file DataSheet2.pdf]

Supplementary Table S2. CGM metrics and Glycemic outcomes with daily UCCS/MCS frequency, with and without patient P2.

A) Descriptive CGM metrics (mean ± SD).

CGM Metric	With Patient P2 (n=10)	Without Patient P2 (n=9)
TBR (%), <70mg/dL [<3.9mmol/L]	11.20 ± 31.24	1.33 ± 1.66
TIR (%), 70–150mg/dL [3.9–8.3mmol/L]	88.10 ± 31.04	97.88 ± 2.47
TAR (%), >150mg/dL [>8.3mmol/L]	0.70 ± 1.16	0.78 ± 1.20
Mean glucose (mg/dL)	92.50 ± 16.39	97.22 ± 7.15
CV (%)	10.62 ± 3.54	10.91 ± 3.62
GMI (%)	4.83 ± 0.61	4.99 ± 0.37
MAD (%)	10.12 ± 2.75	10.21 ± 2.90

B) **Correlation analysis between daily UCCS/MCS** frequency **and annual attack frequency, TBR, TIR, and TAR**

Outcome	With Patient 2 (n=10) ρ (p)	Without Patient 2 (n=9) ρ (p)
Annual atack frequency	-0.854 (p=0.002)	-0.792(p=0.011)
TBR (%)	-0.917 (p=0.001)	-0.879 (p=0.001)
TIR (%)	0.899 (p=0.001)	0.856 (p=0.003)
TAR (%)	-0.337 (p=0.341)	-0.565 (p=0.113)

C) Sensitivity analysis with patient P2 and excluding patient P2: comparison by hypoglycaemia burden (TBR 0% vs ≥2%).

	With PatientP2			Without PatientP2		
Variable	TBR 0%	TBR≥ 2%	p value	TBR 0%	TBR≥ 2%	p value
Attacks/year	0.6 ± 0.55	2.6 ± 0.89	**0.003**	0,6±0.55	2.25±0.5	**0.002**
Daily UCCS/MCS frequency (doses/day)	3.2 ± 0.45	1.6 ± 1.14	**0.019**	3,2±0.45	2±0.82	**0.025**
AST (IU/L)	31.4 ± 4.34	33.6 ± 10.11	0.667	31,4±4.34	36±9.9	0.437
ALT (IU/L)	23 ± 7.97	33 ± 20.14	0.332	23±7.97	38.5±18.41	0.13
Total bilirubin (mg/dL)	0.43 ± 0.06	0.70 ± 0.37	0.154	0,43±0.06	0.72±0.43	0.266
Direct bilirubin (mg/dL)	0.15 ± 0.03	0.28 ± 0.12	0.071	0,15±0.03	0.31±0.13	0.037
GGT (IU/L)	6.8 ± 1.3	12 ± 6.4	0.144	6,8±1.3	13.25±6.65	0.147
Hepatic steatosis on USG, n/N (%)	0/5 (0%)	4/5 (80%)	**0.048**	0/5 (0%)	3/5 (75%)	**0.048**

TBR %0=non-hypoglycemia group; TBR≥2%=hypoglycemia group

%TBR, time below range; UCCS, uncooked cornstarch; MCS, modified cornstarch; USG, ultrasonography. Thresholds for CGM metrics are defined in Methods/Table 1. Continuous variables

are mean ± SD and compared by Student's t-test; proportions by Fisher's exact test (two-sided). Bold p values p<0.05.

D) Abdominal ultrasonography subgroups and outcomes (mean ± SD)

	Abdominal USG*		p-value	Abdominal USG**		p-value
	Normal	**Hepatosteatosis**		Normal	**Hepatosteatosis**	
Attacks/Yr:	0.83±0.75	2.75±0.96	**0.007***	0.83±0.75	2.33±0.57	**0.020**
GMI (%)	5.02±0.26	4.55±0.91	0.385	5.01±0,26	4.93±0.60	0.77
Mean Glucose (mg/dl)	97±6,07	85.75±25.36	0.315	97±6.07	97.66±10.59	0.905
CV%	9,6±1,92	12.15±5.12	0.290	9.6±1.92	13.53±5.27	0.131

*With Patient 2, **Without Patient 2

Notes: Values under A are reported as mean ± SD (from cohort-level tables). Spearman's ρ in B were computed from patient-level data extracted from the manuscript's per-patient table. Outcomes: TBR (%), <70mg/dL [<3.9mmol/L], TIR (%), 70–150mg/dL [3.9–8.3mmol/L], TAR (%), >150mg/dL [>8.3mmol/L]. UCCS/MCS, daily frequency of uncooked/modified cornstarch administration (episodes/day). 'With P2' includes all patients (n=10); 'Without P2' excludes the outlier case (n=9).

USG: Ultrasonography, Attacks/Yr: The number of metabolic attack per year.

GMI: Management Indicator, CV%: coefficient of variation
